# Precision Is Not Enough: When Tools Outpace Translation in Ocular Gene Therapy

**DOI:** 10.3390/genes17030283

**Published:** 2026-02-27

**Authors:** Maram E. A. Abdalla Elsayed, Robert E. MacLaren

**Affiliations:** 1 Nuffield Department of Clinical Neuroscience, University of Oxford, Oxford OX3 9DU, UK; 2 Oxford Eye Hospital, Oxford University Hospitals NHS Foundation Trust, Oxford OX3 9DU, UK

**Keywords:** ocular gene therapy, CRISPR, CRISPR/Cas9, Gene editing, prime editing, base editing, AAV, vector, clinical trials, translation

## Abstract

Advances in molecular biology have positioned the eye as a leading platform for gene therapy, owing to its surgical accessibility, relative immune privilege, and the ability of the contralateral eye to serve as an anatomical control. We trace the historical evolution of gene discovery, synthesize current gene therapy strategies for inherited and acquired ocular disorders, critically evaluating the limitations of CRISPR and related genome-editing technologies, and examine the key scientific and translational challenges that must be addressed for genetic therapies to be integrated into routine ophthalmic practice.

## 1. Introduction

The historical trajectory of biomedical science is marked by distinct eras of discovery, each building upon the last. The Enlightenment sharpened the anatomical gaze. Through systematic dissection and the careful curation of specimens, William and John Hunter, the pioneering anatomists of 18th century Britain, helped convert the body from a theological abstraction into a material system that could be mapped, compared, and repaired, correcting entrenched misconceptions and laying foundations for surgery and modern anatomy [1]. During the 19th and early 20th centuries, scientific emphasis shifted from anatomical structure to physiological function, with physiology emerging as the language of mechanism. This transition was crystallized by conceptual advances such as the Hodgkin–Huxley model of the action potential, which demonstrated that biological processes could be formulated as quantitative, experimentally testable principles rather than purely descriptive narratives. By defining how voltage- and time-dependent ion channel conductances generate and propagate nerve action potentials, the model provided a biophysical framework for neuronal excitability and transformed neural signaling into a mechanistically grounded, experimentally testable discipline [2]. If anatomy taught us what is there, and physiology taught us how it works, the 21st century is teaching us something more intimate: why it differs from one individual to the next.

That “why” is increasingly genetic. Understanding this genetic “why” is precisely what drives the development of gene therapies: by identifying the causal variants behind individual differences, we can design interventions that correct the underlying defect or compensate for its functional consequences. Building on the shift from descriptive anatomy to mechanistic physiology—and now to genotype-defined causality—ocular therapeutics have similarly progressed from structural repair to molecular correction at the level of the underlying mutation. High-throughput sequencing, genome-wide association studies, and the post-Human Genome Project ecosystem of tools have made connecting nucleotide-level differences to phenotypes across populations and across the lifespan possible at an unprecedented scale [3,4,5]. Ophthalmology is uniquely poised to capitalize on this revolution in a manner unmatched by most organ systems. Anatomically, the retina functions as a discrete extension of the central nervous system, enclosed within an immune-privileged environment that mitigates inflammatory reactivity and thereby improves the tolerability of gene and cell-based interventions. This compartmentalization allows targeted delivery of vectors or therapeutics to well-defined tissues—such as the retinal pigment epithelium, photoreceptors, or trabecular meshwork—while limiting systemic exposure. At the same time, no other neural tissue permits such high-resolution, longitudinal phenotyping through non-invasive modalities: optical coherence tomography, adaptive optics imaging, and widefield angiography render cellular and microstructural consequences of genetic variation visible in vivo. The eye therefore offers a rare opportunity to correlate genotype with dynamic phenotype in real time, enabling investigators to observe how molecular perturbations affect cell biology, tissue architecture, and functional vision.

This article traces the historical evolution of gene discovery and its application to ocular disease, synthesizes current gene therapy strategies for both inherited and acquired ocular disorders, and provides a critical evaluation of CRISPR and related genome-editing technologies, with particular emphasis on their technical, biological, and safety-related limitations. In addition, it examines the major scientific, clinical, and translational challenges that must be addressed to enable the effective, safe, and scalable integration of genetic therapies into routine ophthalmic practice.

## 2. Evolution of Ophthalmic Genetics

The eye represents an especially strong candidate for gene therapy due to the extensive characterization of its genetic landscape and the high prevalence of monogenic, well-phenotyped ocular disorders. The modern era of ophthalmic genetics began in the late 1980s and early 1990s when linkage analysis and positional cloning identified the first disease genes. The rhodopsin gene (*RHO*) on chromosome 3q22.1 was the first gene linked to autosomal dominant retinitis pigmentosa in 1989–1990; soon afterwards mutations in *RHO* were shown to cause both dominant and recessive retinitis pigmentosa [6,7]. In the same year the gene responsible for choroideremia was mapped and cloned [8]. These discoveries ended decades of speculation about the aetiology of inherited retinal degenerations and demonstrated that one gene could produce diverse phenotypes. Progress accelerated with the Human Genome Project and the development of next-generation sequencing (NGS), which enabled comprehensive mutation screening [9,10,11]. Since the first *RHO* mutation was described in 1990, more than 400 genes and loci have been recognized as causative for retinal disease [12]. Genetic heterogeneity is particularly striking in retinitis pigmentosa and many genes can produce multiple phenotypes; for example, variants in *PRPH2* can cause autosomal dominant retinitis pigmentosa, autosomal dominant macular dystrophy or more rarely, digenic retinitis pigmentosa due to combined variants in *PRPH2* and *ROM1* [13,14].

The expansion of gene discovery has not been limited to retinal dystrophies. In glaucoma, *MYOC* was identified in 1997 as the first gene causing primary open-angle glaucoma (POAG) and encodes myocilin, a protein believed to have a role in cytoskeletal function. Mutations in *MYOC* are linked to the GLC1A locus and over 100 POAG-associated mutations have been identified in the *MYOC* gene making it accountable for 3–5% of POAG cases worldwide and often result in juvenile or early-adult disease requiring surgical management [15]. Subsequently, *OPTN* on chromosome 10 was discovered; its E50K variant is associated with normal-tension glaucoma and leads to severe phenotypes despite normal intraocular pressure [15]. *OPTN* encodes optineurin, a coiled-coil–containing adaptor protein implicated in vesicular transport and secretory pathway dynamics [16]. At the cellular level, optineurin localizes to the Golgi complex and interfaces with trafficking machinery, contributing to Golgi integrity, membrane trafficking, and regulated exocytosis [17]. Pathogenic *OPTN* variants have been linked to primary open-angle glaucoma, and are proposed to promote retinal ganglion cell vulnerability through disrupted intracellular trafficking and stress-response pathways, consistent with a role for impaired proteostasis and axonal homeostasis in glaucomatous neurodegeneration [18,19]. WD repeat domain 36 (WDR36) mutations were initially reported but later studies questioned their pathogenicity [20]. The identification of glaucoma genes not only informed disease pathogenesis but also highlighted that common ocular disorders have significant genetic contributions.

In age-related macular degeneration (AMD), initial observations of familial aggregation and increased risk in relatives led to early candidate gene studies, notably implicating the *APOE* gene, but the first major breakthroughs came from linkage analyses that identified susceptibility loci at 1q31 (complement factor H, CFH) and 10q26 (ARMS2/HTRA1), which together account for a significant proportion of AMD heritability [21,22]. Subsequent genome-wide association studies (GWAS) and meta-analyses expanded the number of implicated loci, with large-scale analyses identifying over 30–34 loci and more than 50 independently associated variants that influence AMD risk; importantly, many signals map to non-coding regions, consistent with a major contribution of gene-regulatory mechanisms [23,24,25]. The identification of risk alleles at the CFH (chromosome 1q31) and ARMS2/HTRA1 (chromosome 10q26) loci has been central to understanding the heritability and pathogenesis of age-related macular degeneration. Large-scale genome-wide association studies and meta-analyses have consistently shown that these loci account for a substantial proportion of AMD risk. Risk variants in CFH implicate dysregulated alternative complement pathway control in AMD, consistent with reduced local inhibition of C3 activation at the RPE–Bruch’s membrane interface and a shift toward chronic complement activation [26,27,28]. In parallel, variants at the 10q26 ARMS2/HTRA1 locus are linked to pathogenic remodeling of the extracellular matrix and pro-angiogenic disease biology, supported by genetic association with neovascular AMD and experimental evidence that increased HTRA1 activity can alter Bruch’s membrane ultrastructure via cleavage of extracellular matrix components [29,30,31]. The CFH Y402H polymorphism compromises factor H recruitment to Bruch’s membrane by reducing its affinity for heparan sulphate proteoglycans, thereby weakening local control of the alternative complement pathway (i.e., less efficient regulation of C3b deposition and C3 convertase activity). This loss of surface-directed complement inhibition is expected to favour chronic complement activation at the RPE–Bruch’s membrane interface and to promote sub-RPE deposit (drusen/basal deposit) accumulation, linking a defined biochemical binding defect to hallmark early AMD pathology [32,33,34]. In contrast, the ARMS2/HTRA1 locus, though functionally complex due to high linkage disequilibrium, is increasingly understood to affect HTRA1 expression in the retinal pigment epithelium, impacting extracellular matrix integrity and promoting choroidal neovascularization [35,36]. Risk alleles at CFH and ARMS2/HTRA1 have been found to be associated with increased risk and earlier onset of late AMD, particularly choroidal neovascularization for ARMS2/HTRA1 [23,37,38,39]. Moreover, homozygous carriers of ARMS2/HTRA1 risk alleles have markedly elevated odds ratios for geographic atrophy and neovascular AMD, with diagnosis occurring nearly a decade earlier than non-carriers [23]. CFH risk alleles have additionally been shown to accelerate progression to late-stage disease, but the phenotypic spectrum and response to anti-VEGF therapy may differ between CFH- and ARMS2/HTRA1-driven AMD [40]. Advances in multimodal retinal imaging, including optical coherence tomography (OCT) and fundus autofluorescence, have enabled more precise genotype-phenotype correlations and earlier detection of atrophic changes, facilitating tailored monitoring strategies for high-risk individuals. Additionally, polygenic risk scores incorporating CFH, ARMS2/HTRA1, and other loci have been developed to estimate individual risk and stratify patients, but their clinical utility remains limited due to challenges in interpretation and lack of prospective validation [41]. Nevertheless, the presence of CFH and ARMS2/HTRA1 risk alleles is strongly associated with increased lifetime risk and faster progression rates to late AMD [38]. For example, homozygous ARMS2/HTRA1 carriers have a cumulative lifetime risk of late AMD approaching 27%, compared to 4% in non-carriers [42]. Moreover, emerging evidence indicates that systemic levels of complement factor H-related proteins (FHRs), which are influenced by CFH locus variants, further refine risk stratification and may help identify individuals most likely to respond to targeted therapeutic approaches [38].

## 3. Unique Advantages of the Eye for Gene Therapy

The eye offers several unique anatomical, physiological, and immunological features that make it an exceptionally attractive target for gene therapy. The human eye is only ~24 mm in length and is divided into discrete compartments (anterior chamber, vitreous cavity and subretinal space) [43]. This compact and compartmentalized structure allows gene therapy vectors to be delivered in low volumes and to stay confined within a defined space, minimizing systemic exposure. Subretinal injection places vectors directly under the retina and provides intimate contact with target photoreceptors and retinal pigment epithelium (RPE), while intravitreal injection delivers into the vitreous cavity and can reach the inner retina [44]. Suprachoroidal and corneal intrastromal routes provide additional options for targeting choroid and cornea [45,46]. As the eyes are superficially located, they are readily accessible via minimally invasive techniques; ophthalmic surgeons routinely perform pars plana vitrectomy or intravitreal injection under local anaesthesia, allowing precise vector delivery. Non-integrating vectors can be administered without major systemic risk, and in bilateral ocular diseases the fellow eye can serve as an anatomical control.

Beyond its compartmentalized structure, ocular tissues are optically clear, enabling non-invasive imaging modalities such as fundus photography, OCT, fluorescein/indocyanine angiography and fundus autofluorescence. These techniques provide high-resolution structural and functional information and allow longitudinal monitoring of gene therapy effects. The retina can be visualized repeatedly to assess vector distribution, gene expression (via reporter fluorescence), and retinal thickness and function [47]. Functional outcomes can be measured with microperimetry [48]. The ability to monitor both efficacy and safety in real time is a unique advantage over gene therapies in other organs.

The post-mitotic nature of photoreceptors and retinal pigment epithelial cells is a critical advantage for achieving durable gene expression, as these non-dividing cells can retain episomal viral vectors for extended periods without the dilution of transgene expression that occurs with cell division [49]. Non-integrating vectors such as adeno-associated virus (AAV) deliver genes in episomal form; because the target cells do not divide, the vector genomes are not diluted, leading to durable expression [50,51,52]. Successful AAV gene therapies, e.g., Luxturna and Zolgensma, target cells with low turnover and RPE cells have extremely low turnover rates, enabling therapy to remain effective for 3–7.5 years [53,54,55,56]. This characteristic enables a single administration of gene therapy to provide long-term, potentially lifelong, therapeutic benefit, as demonstrated by the sustained efficacy observed in clinical trials of voretigene neparvovec for RPE65-associated retinal dystrophy [53,54]. Unlike hepatocytes or hematopoietic cells, ocular target cells do not proliferate extensively, reducing risk of insertional mutagenesis and enabling non-integrating vector use.

The eye possesses a distinctive immunological milieu that supports gene therapy by limiting inflammatory responses and facilitating sustained transgene expression [57,58]. Central to this environment are the blood-ocular barriers and restricted lymphatic drainage. Tight junction complexes form the blood-retina barrier at the level of the retinal vascular endothelium and the blood-aqueous barrier within the ciliary epithelium and iris vasculature, effectively isolating intraocular compartments from systemic circulation and minimizing exposure to peripheral immune surveillance [57,58]. In parallel, the avascularity of the cornea and the relative absence of classical lymphatic channels reduce antigen presentation and lymphocyte recruitment, thereby contributing to the designation of the eye as an immune-privileged site capable of tolerating foreign antigens with attenuated systemic response [59,60]. Within the retina, immunomodulatory mediators, such as transforming growth factor-β, α-melanocyte-stimulating hormone, and vasoactive intestinal peptide, alongside membrane-bound regulators including complement inhibitors, Fas ligand, and PD-L1, actively suppress pro-inflammatory cascades and limit activation of effector leukocytes [61]. These molecular and anatomical features operate synergistically to sustain ocular immune privilege.

This immune deviation is further reinforced by anterior-chamber-associated immune deviation (ACAID), a form of peripheral tolerance elicited when antigens enter the anterior chamber or vitreous. Antigen-presenting cells conditioned within the ocular microenvironment traffic to secondary lymphoid organs, where they promote the induction of regulatory T cells and dampen conventional T-cell effector pathways [62]. The resulting systemic tolerance mitigates tissue-damaging inflammation and is thought to support long-term acceptance of viral vectors and transduced cells in the context of gene therapy [57,58]. In the open-label, multicenter Phase II GEMINI study evaluating bilateral sequential subretinal administration of timrepigene emparvovec in adults with genetically confirmed choroideremia, systemic immunological safety was a key outcome measure. The study demonstrated an acceptable systemic safety profile over 12 months of follow-up, with no evidence of clinically significant systemic immune activation following gene transfer [63]. Importantly, comprehensive immunogenicity assessments, including ELISpot assays to detect peripheral T-cell responses against the AAV2 vector or REP1 transgene, revealed an absence of sustained systemic cellular immunity attributable to vector exposure [62]. This finding indicates that subretinal delivery of timrepigene emparvovec did not elicit deleterious systemic effector responses, a result consistent with the eye’s relative immunological privilege and containment of vector-associated antigens within the ocular microenvironment. The lack of long-term systemic immune reactivity supports the tolerability of bilateral ocular gene therapy in choroideremia and informs future strategies for sequential treatment of other ocular diseases.

Nonetheless, ocular immune privilege is relative rather than absolute. Barrier disruption, excessive vector dosing, or pronounced innate and adaptive immune activation can overcome local suppression and precipitate inflammation [57,58,64]. Subretinal administration may activate resident microglia and macrophages, while intravitreal delivery exposes vectors to neutralizing antibodies and complement, with the potential to diminish transgene expression and compromise therapeutic durability [44,58]. Clinical experience has shown that gene therapy-associated uveitis may occur; however, early intervention with corticosteroid results in favourable outcomes [65]. These observations underscore that although the ocular environment is comparatively permissive to gene transfer, immune privilege has practical limits that must be considered. The severity of immune and inflammatory reactions is influenced by multiple factors, including vector serotype, dose, route of administration (subretinal versus intravitreal), vector construct design, and patient demographics, with subretinal delivery generally producing a weaker humoral response but potentially stronger local inflammation compared to intravitreal delivery [66]. Ongoing research is focused on mitigating these immune responses through strategies such as capsid engineering, immunosuppression regimens, dose optimization, and the development of less immunogenic vector constructs to enhance the safety and durability of ocular gene therapy [67]. Despite these constraints, the immune-modulating properties of the eye confer clear therapeutic advantages and permits unilateral treatment, with the contralateral eye serving as an internal control for assessing therapeutic efficacy and safety.

## 4. CRISPR and Genome Editing in Ophthalmology

Most current ocular gene therapies employ gene replacement or augmentation strategies, whereby a functional copy of the absent or defective gene is introduced into target cells to restore physiological protein expression. This approach is particularly applicable to loss-of-function mutations in monogenic retinal disorders, provided the therapeutic payload is sufficiently small to be accommodated within the ∼4.6 kb packaging capacity of the icosahedral adeno-associated virus (AAV) capsid [68]. In such contexts, gene replacement offers a mechanistically direct intervention, compensating for the defective allele by supplying exogenous gene product. However, this strategy is inherently constrained by disease mechanism and vector capacity: it is ineffective for gain-of-function or dominant-negative mutations, which instead necessitate gene silencing or editing modalities, and genetically heterogeneous conditions require the development of distinct constructs for each implicated gene, limiting scalability. Many inherited retinal disease genes (e.g., *ABCA4* at 6.8 kb, *MYO7A* at 7.5 kb, *USH2A* at 15.7 kb) exceed this capacity [67]. Investigators have explored dual-AAV systems to reconstitute large genes in situ or have turned to alternative vectors, but efficient and consistent reassembly remains challenging [69,70,71]. Table 1 summarises the principal therapeutic modalities currently under investigation.

**Table 1:** Gene therapy approaches and characteristics.

Approach	Description	Advantages	Limitations	Suitable for
Gene replacement/augmentation (Figure 1)	Delivery of a functional cDNA copy to cells via viral or non-viral vectors	Simple concept; effective for recessive loss-of-function mutations; can provide long-term expression	Requires knowledge of causative gene; cannot treat gain-of-function mutations; limited by vector capacity; expensive to develop individual therapies	Monogenic recessive disorders, e.g., RPE65-LCA, GUCY2D-LCA, MERTK-RP
Gene silencing (RNAi/antisense) (Figure 2)	Use of siRNA or antisense oligonucleotides to degrade mutant mRNA	Exploits natural RNA interference; can suppress dominant mutations	RNA instability; poor bioavailability; off-target effects and immunogenicity	Dominant mutations; diseases involving overexpressed proteins such as autosomal dominant RHO mutations
Gene editing (CRISPR, base editing, prime editing)	Correction of specific nucleotide changes using programmable nucleases or editors	Potentially permanent correction; can address dominant and recessive mutations; precision editing (base/prime editors) avoids DSBs	Off-target effects; low HDR efficiency in post-mitotic cells; delivery of large editors is challenging; long-term safety unknown	Monogenic disorders with point mutations (base/prime editing); gene disruption for dominant alleles; conditions with genes too large for augmentation (e.g., CEP290)
Modifier gene therapy	Delivery of genes that modulate disease pathways (e.g., transcription factors)	Mutation-agnostic; one therapy can treat multiple genotypes	Identifying appropriate modifiers is challenging; risk of pleiotropic effects	Polygenic disorders; diseases with multiple causative genes

**Figure 1:**
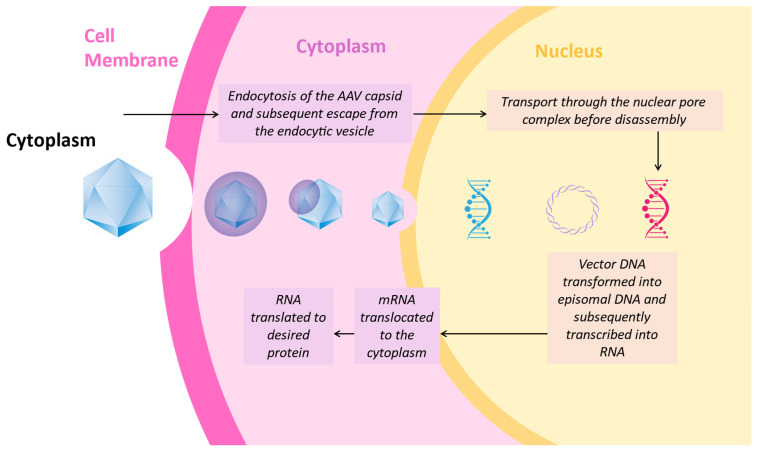
AAV-Mediated Gene Augmentation. Intracellular Trafficking and Episomal Expression Following AAV Gene Transfer.

**Figure 2:**
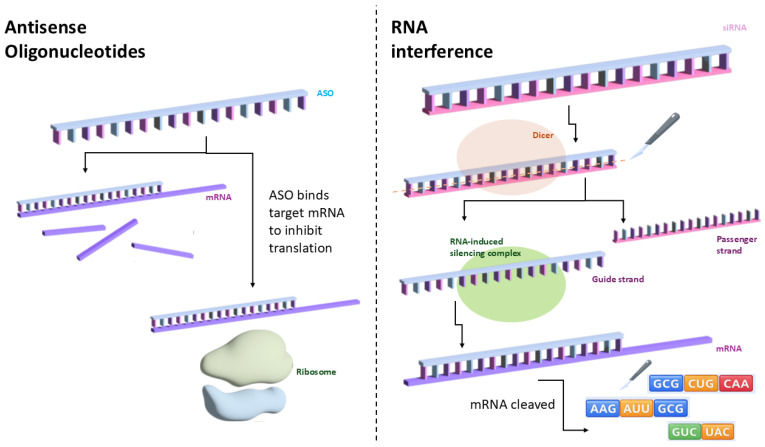
Gene silencing. Gene-silencing therapies act to stop the translation of mRNA via the use of antisense oligonucleotides (ASOs) or small interfering RNA (siRNA) which are double-stranded non-coding RNA molecules.

Gene silencing strategies, including small interfering RNA (siRNA) and antisense oligonucleotides, aim to attenuate expression of pathogenic transcripts or proteins [72]. While conceptually suited to dominant or gain-of-function conditions, these agents face practical challenges, including limited intracellular stability, suboptimal bioavailability, and the potential for off-target effects or innate immune activation [73]. Emerging approaches for neovascular age-related macular degeneration demonstrate evolving design complexity; for example, 4D-150 incorporates microRNA-based suppression of VEGF-C alongside anti-VEGF expression to enhance therapeutic breadth [74].

Modifier gene therapy constitutes an alternative paradigm in which a gene is delivered not to replace a specific defective sequence, but to modulate downstream pathways and improve cellular resilience across multiple genotypes. This mutation-agnostic strategy offers theoretical advantages for genetically diverse diseases; OCU400 exemplifies this concept, delivering the transcription factor NR2E3 to reprogram photoreceptor homeostasis and promote phenotype stabilisation irrespective of the causative mutation [75]. NR2E3 is a photoreceptor-specific nuclear receptor transcription factor that functions during retinal development to promote rod fate while repressing cone gene programmes, thereby shaping rod embryogenesis and maturation [76,77]. Pathogenic NR2E3 variants disrupt this regulatory network, leading to aberrant photoreceptor specification and/or mis-expression of rod and cone transcripts in differentiated cells. The resulting imbalance in photoreceptor identity destabilizes core transcriptional programs required for outer segment maintenance, metabolic homeostasis, and phototransduction, leading to aberrant co-expression of rod and cone gene networks [78]. This transcriptional dysregulation imposes chronic cellular stress by overwhelming protein quality-control pathways, perturbing energy homeostasis, and compromising outer segment renewal, thereby rendering photoreceptors increasingly vulnerable to oxidative stress and cumulative damage, ultimately driving retinal degeneration [78,79]. Collectively, these developments illustrate a therapeutic landscape progressing beyond traditional replacement strategies toward pathway-directed and genotype-independent intervention.

The CRISPR/Cas9 system from *Streptococcus pyogenes* has revolutionised genome editing [80]. It consists of a programmable guide RNA (gRNA) directing the Cas9 nuclease to a specific DNA sequence adjacent to a protospacer adjacent motif (PAM) [80,81]. Cas9 induces a double-strand break (DSB) that is repaired by non-homologous end joining (NHEJ) or homology-directed repair (HDR) [81]. In retinal cells homology-directed repair is inefficient, leading to unpredictable indels and potential large deletions or chromosomal rearrangements [82,83]. Nonetheless, CRISPR/Cas9 can disrupt dominant disease alleles or correct recessive mutations [84]. Table 2 summarises the main gene editing modalities. The first in vivo CRISPR trial in ophthalmology, EDIT-101, used an AAV5 vector to deliver two gRNAs and SpCas9 to edit the CEP290 gene in LCA10 [85]. The CEP290 gene (8 kb) cannot fit in AAV; thus, gene augmentation is not feasible [86]. Early results from the BRILLIANCE trial reported no serious adverse events or dose-limiting toxicities [85]. Although the current trial enrolled only a small cohort, ongoing follow-up will be essential to assess the long-term durability of editing and the potential for off-target effects [85,87]. While Editas Medicine has paused further enrolment because of a limited responder population and uncertain commercial viability, the clinical experience to date indicates that CRISPR-mediated editing is safe in the human eye and provides an encouraging foundation for the continued refinement and expansion of genome-editing approaches for retinal disease [85,87].

### 4.1. Base Editing

Base editors couple dCas9 or Cas9 nickase to a cytidine or adenine deaminase, enabling direct C → T or A → G conversions without DSBs [88,89]. The editing window spans ~5 nucleotides, and bystander nucleotides within this window may also be edited [88]. Base editing is attractive for correcting single-nucleotide variants that constitute the majority of pathogenic variants [89]. In post-mitotic retinal cells, base editing yields higher editing efficiency than HDR [90,91]. For example, an in vivo base editing achieved up to 44% editing of the rhodopsin E150K mutation in mice with minimal off-target effects [92]. Another study used base editing to correct the RPE65 c.130C>T mutation in vivo in mice; editing efficiency reached ~29% and restored visual function [93]. Muller et al. report the development of a dual-AAV split-intein adenine base editor that efficiently corrects the common *ABCA4* c.5882G>A (p.Gly1961Glu) mutation across human retinal models, mice, and nonhuman primates, achieving high in vivo editing rates in cones and RPE cells without detectable off-target effects, thereby demonstrating strong translational potential for base editing in Stargardt disease and other retinal disorders. [94]. Limitations include off-target deamination at mismatched sites and bystander edits; improved deaminases and guide design can mitigate these issues [89,95].

### 4.2. Prime Editing

Prime editing uses a Cas9 nickase fused to reverse transcriptase and a pegRNA containing both the guide sequence and a template specifying the desired edit [96]. It can perform all possible base changes, small insertions and deletions, and does not rely on DSBs or donor templates [96]. A recent study applied a dual-AAV prime editing system to correct the Pde6b Y347X nonsense mutation in rd1 mice [97]. The editing efficiency was 26.47 ± 13.35%, and genome-wide off-target analysis showed minimal off-target editing [97]. Treated mice exhibited restored PDE6B protein expression and improved visual function [97]. This proof-of-concept demonstrates the feasibility of prime editing in photoreceptors despite the large size of the prime editor. However, the prime editor (~6.3 kb) exceeds the AAV payload; the investigators split Cas9 into two AAV vectors and reconstituted it via an intein-mediated trans-splicing mechanism [97]. Such dual-vector strategies complicate manufacturing and may reduce co-transduction efficiency. Prime editing efficiency in human photoreceptors remains unknown, and off-target evaluation must consider both insertions/deletions and reverse transcriptase template switching [96,98]. Moreover, prime editing remains predominantly evaluated in preclinical models of recessive disease, and its capacity to correct dominant gain-of-function mutations has yet to be demonstrated in vivo. A further translational challenge lies in the physical size of current editing constructs; prime and base editors exceed the packaging limits of conventional AAV vectors, necessitating the development of more compact editing architectures or alternative delivery platforms (e.g., dual-vector systems or non-viral nanoparticles). Continued optimisation in compact editor design and delivery will be essential to enable efficient, clinically scalable delivery in ocular gene editing applications [97,99].

**Figure 3:**
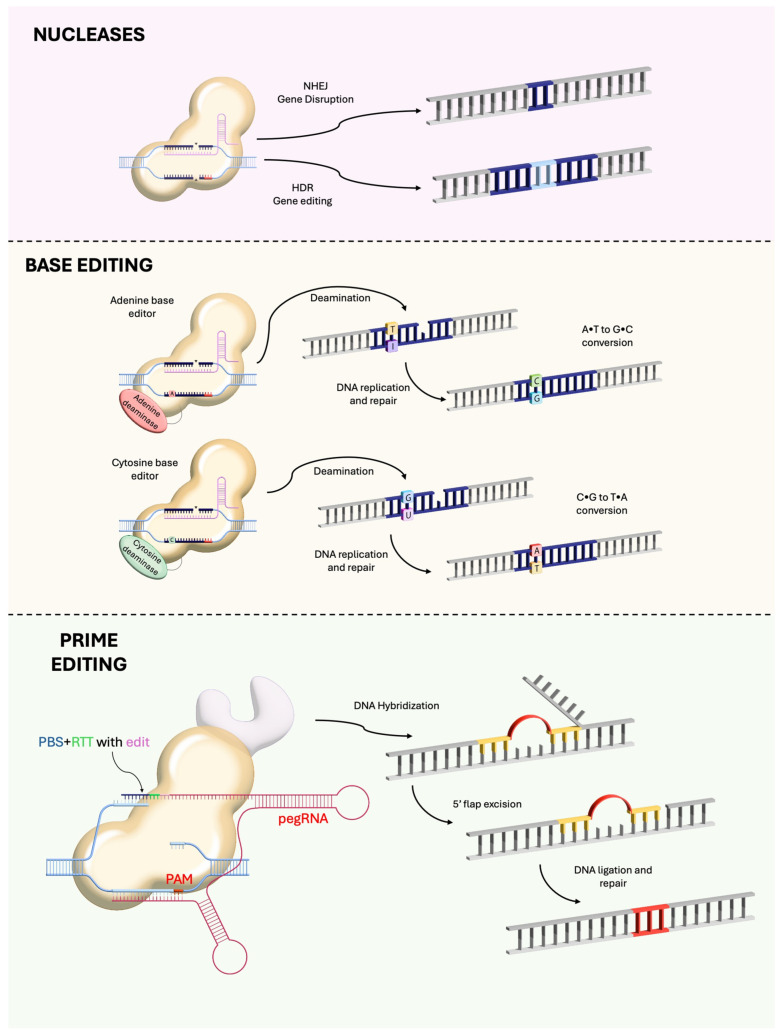
Three classes of CRISPR-Cas-derived genome editing agents—nucleases, base editors, and prime editors. NHEJ = non-homologous end joining; HDR = homology-directed repair; PBS = primer-binding site; RTT = reverse transcription template; PAM = protospacer adjacent motif. The PegRNA consists of an extended single guide RNA (sgRNA) containing a primer binding site and a reverse transcriptase.

### 4.3. Beyond Monogenic Diseases

Although inherited retinal degenerations represent an optimal entry point for ocular gene therapy due to their monogenic aetiology and well-defined molecular targets, the broader clinical and societal impact of these technologies will ultimately depend on successful translation to common, complex blinding diseases such as glaucoma, age-related macular degeneration and diabetic retinopathy [100,101]. Choroidal neovascularisation (CNV) underlying neovascular AMD is driven by overproduction of VEGF [102]. Anti-VEGF biologics require frequent intravitreal injections and carry risks of endophthalmitis or systemic adverse events [103]. CRISPR offers gene-level VEGF suppression. Chung et al. used lentiviral vectors delivering Cas9 and VEGFA-targeting guide RNAs to the subretinal space; the treatment reduced VEGF-A expression, lowered neovascular lesion area and preserved retinal function in rodent models [104]. However, gene knockdown of VEGF may induce compensatory up-regulation of VEGF family members and could perturb normal angiogenesis [105]. Alternative strategies include genome editing of pro-angiogenic regulators such as HIF or complement components [106].

Viral vectors face cargo-size limitations and immunogenicity; therefore, non-viral nanoparticles are being developed to deliver CRISPR components as mRNA or ribonucleoprotein complexes [107]. Cao and colleagues synthesised iminoboronate ester-based lipidoids that form dynamic covalent bonds with aldehyde-containing molecules. These lipidoids were formulated into LNPs encapsulating Cas9 mRNA and single-guide RNA (sgRNA) targeting VEGFA. Intravitreal injection of the LNPs into laser-induced CNV mice resulted in efficient delivery to retinal pigment epithelium (RPE) cells. The LNPs disrupted VEGFA and markedly reduced CNV areas, outperforming anti-VEGF drugs [108]. The design leverages reversible covalent linkages that undergo H_2_O_2_-triggered cleavage under oxidative conditions to release CRISPR cargo. Importantly, the LNPs penetrated the neural retina via Müller cell transcytosis, enabling delivery throughout the outer retina [108]. Another strategy was used by Gautam et al. who engineered charge-variant lipid nanoparticles incorporating amine-modified (LNPa), carboxyl-ester–modified (LNPx), carboxyl-modified (LNPz), or unmodified PEG-lipids and demonstrated that, following subretinal delivery, LNPa and conventional LNPs predominantly transduced the retinal pigment epithelium, whereas LNPx and LNPz unexpectedly mediated substantial photoreceptor transfection (27% and 16%, respectively) [109]. This cross-disciplinary innovation from organic chemistry and nanomedicine demonstrates how material-responsive carriers can improve ocular gene editing. These studies show that non-viral delivery can achieve therapeutically relevant editing efficiencies while avoiding integration risks and packaging constraints. Looking ahead, integration of gene-based interventions with emerging tools such as polygenic risk stratification may enable a precision medicine framework capable of addressing the leading causes of irreversible blindness worldwide.

Primary open-angle glaucoma (POAG) is often associated with mutations in MYOC or dysregulation of aqueous humour outflow [110]. In a landmark preclinical study, AAV-delivered CRISPR selectively disrupted the mutant MYOC allele in human and mouse trabecular meshwork cells. Gene editing reduced misfolded myocilin accumulation, preserved trabecular meshwork morphology and prevented elevation of intraocular pressure (IOP) [111]. Another approach targeted Aquaporin 1 (AQP1) expressed in ciliary body epithelia: AAV-mediated CRISPR knockout of Aqp1 lowered IOP in mice [112]. Together, these experiments highlight the potential of CRISPR to modulate genes controlling aqueous humour dynamics.

Emerging RNA-targeting editors such as high-fidelity Cas13d (hfCas13d) enable transient knockdown of mRNAs encoding AQP1 or carbonic anhydrase (CA2) without permanent DNA modification, while maintaining on-target efficiency comparable to wild-type Cas13d [113]. Disruption of these transmembrane proteins in the mouse ciliary body results in reduced intraocular pressure. Consistent with this mechanism, intravitreal delivery of hfCas13d mRNA packaged in AAV significantly lowered intraocular pressure in corticosteroid-induced glaucoma models and in wild-type mice, with effects persisting for several months [113,114]. By targeting RNA rather than DNA, Cas13-based approaches avoid permanent genomic alterations and mitigate risks associated with off-target DNA cleavage.

## 5. An Expanding Toolkit, Limited Cures: Translational Bottlenecks in Gene Editing

Gene editing has become a field in which conceptual elegance routinely outpaces clinical translation. In barely a decade, the academic community has assembled a sprawling toolkit: programmable nucleases (Zinc Finger Nucleases, TALENs and CRISPR-Cas systems), base editors, prime editors, and an expanding menagerie of delivery vehicles yet the number of gene-editing therapies in clinical trials remains small relative to the breadth of genetic disease [115,116,117]. This disparity is often narrated as a temporary lag between discovery and translation. We argue that it reveals a structural misalignment in how we reward invention, how we evaluate risk, and how we organize the practical work of turning molecular interventions into care [118].

The case for a genuinely advancing field is justified. The approval of Casgevy (exagamglogene autotemcel) for sickle cell disease marked the first regulatory endorsement in the United States of a CRISPR-based therapeutic modality, and it built on years of clinical experience showing that editing hematopoietic stem cells can yield sustained clinical benefit in hemoglobinopathies [119,120]. From a scientific vantage point, this is not a marginal milestone: it confirms that genome editing can be engineered, manufactured, deployed, and regulated as a medicine. Parallel advances in ocular gene therapy—exemplified by voretigene neparvovec (Luxturna) for RPE65-associated retinal dystrophy and the Phase III STAR trial in choroideremia, which met key secondary endpoints—indicate that genetic therapies can confer meaningful improvements in visual function when clinical endpoints are coherently matched to the therapeutic mechanism [121,122]. The apparent scarcity of cures is therefore not a verdict against gene editing; it is the predictable early-stage signature of an emerging therapeutic class

The sceptical case, however, is not merely impatience; it reflects a recognition that early successes in gene editing have clustered around clinical contexts that are unusually tractable. Within ophthalmology, voretigene neparvovec benefited from a confluence of favourable translational conditions: subretinal AAV2 delivery reliably targets the retinal pigment epithelium; a naturally occurring RPE65-deficient canine model enabled large-eye dose-ranging and safety evaluation; the human disease, while early-onset, severe and progressive, often retains sufficient retinal structure to define a therapeutic window; and efficacy could be anchored to a validated performance-based functional endpoint, the multi-luminance mobility test (MLMT), accepted by FDA in the pivotal programme [54,121,123]. An additional—and biologically compelling—argument for why Luxturna proved unusually “endpoint-legible” is that RPE65 sits at a mechanistically proximal point in the retinoid visual cycle, so restoring its activity should yield relatively direct, interpretable gains in low-luminance vision. RPE65 is an RPE-expressed isomerohydrolase that enables regeneration of 11-cis-retinoids required for photopigment reconstitution; loss of function produces chromophore starvation with disproportionately severe defects in rod-mediated sensitivity and dark adaptation that can precede extensive anatomic loss, making changes in light sensitivity a meaningful readout of therapeutic rescue [124,125,126]. This biology explains why low-luminance performance measures were unusually informative in the program: the multi-luminance mobility test could be argued to reflect real-world function under graded dim illumination, while full-field stimulus threshold (FST)—a whole-field psychophysical assay designed for very low vision and unstable fixation—quantified global light sensitivity in a way that remains measurable when standard acuity and perimetry are insensitive or infeasible [127]. Consistent with this alignment, FST was prespecified as a secondary endpoint in the Luxturna registration package and showed substantial sensitivity improvements compatible with restoration of chromophore supply rather than marginal shifts in high-contrast acuity [128]. Importantly, this tight coupling between mechanism and measurement cannot be readily extrapolated to many other retinal dystrophies.

This is precisely where the field’s internal debate becomes most important. A prevailing line of reasoning contends that we still need new tools: editors with higher fidelity, lower immunogenicity, and more predictable repair outcomes; and delivery systems with acceptable therapeutic indices. The empirical record supports this argument. Although CRISPR-based genome editing has transformed molecular biology by enabling targeted manipulation of DNA with unprecedented ease, its performance is far from uniform across different mutations. The apparent precision of the platform is constrained by target recognition and by the genomic environments in which mutations reside, such that two variants differing by a single nucleotide can present markedly different levels of editability [129,130,131]. A primary factor impacting CRISPR’s efficacy is the requirement for a protospacer adjacent motif (PAM). The most used Cas9, derived from *S. pyogenes*, recognizes a specific PAM sequence (usually NGG) that is crucial for its binding and subsequent cleavage of DNA [129]. Mutations that lie within, or immediately adjacent to, PAM-proximal regions can therefore be intrinsically refractory to editing: disruption of PAM recognition diminishes nuclease engagement, while subtle sequence changes near the cut site can compromise guide pairing and reduce cleavage efficiency, a problem that becomes particularly acute for gain-of-function alleles [96,130,131]. Active efforts are addressing current targeting and repair constraints through the engineering and discovery of CRISPR nucleases with expanded PAM compatibility, thereby increasing the accessible genomic target space. In parallel, advances in delivery modalities and template presentation are being pursued to enhance precise editing outcomes—particularly in non-dividing cells where HDR is inefficient—alongside HDR-independent precision approaches such as base and prime editing. Engineering efforts have broadened the PAM repertoire through variants such as xCas9 and near-PAMless systems like SpRY, yet these innovations do not abolish context dependence; expanded targeting can introduce new trade-offs in activity and specificity, and certain loci remain difficult because the structural and sequence constraints that shape R-loop formation and nuclease kinetics are not fully relaxed by altered PAM compatibility [132,133].

Equally consequential are epigenetic and chromatin determinants of accessibility, which impose a regulatory layer beyond nucleotide sequence. The chromatin state significantly impacts gene accessibility; regions of heterochromatin are more compact and less accessible to CRISPR components, leading to reduced editing efficiency compared to euchromatic regions [134]. For mutations situated in heterochromatic areas, the likelihood of successful CRISPR-mediated editing is substantially decreased, complicating the process of targeting specific genetic alterations within those domains. Moreover, the presence of DNA methylation can further inhibit CRISPR activity. DNA methylation, typically the addition of a methyl group to cytosines at CpG sites, is a fundamental epigenetic mechanism that has evolved in non-human primates, modulating gene expression without altering DNA sequence. Divergent, tissue-specific methylation at regulatory elements (e.g., promoters and enhancers) can reshape gene regulatory programs and contribute to phenotypic variation in domains such as neurodevelopment, immunity, and metabolism. It has been demonstrated that DNA methylation patterns can directly influence the binding capability of guide RNA and Cas9, thus affecting the overall efficacy of CRISPR editing [134].

Even when cleavage is achieved, the final genotype is governed by endogenous DNA repair pathways: non-homologous end joining predominates in most cells and frequently yields heterogeneous insertions and deletions that are well suited to knockout strategies but ill-suited to precise correction of point mutations, whereas homologous recombination—while capable of templated precision—is restricted by cell-cycle state, template availability, and generally lower efficiency in many cell types [135,136]. Moreover, repair is not uniform along the protospacer; sequence context near the PAM and local microhomology can bias end-joining products, that erode predictability when mutations occur in sensitive PAM-proximal windows [137]. Thus, the challenge of editing “difficult” mutations is not a single bottleneck but the convergence of PAM-imposed geometry, chromatin-mediated accessibility, and repair pathway dynamics, underscoring why continued improvements in nuclease engineering, target design, and strategies that reprogram repair remain essential to realizing the full potential of CRISPR for precise genome modification. Toolmaking, on this account, is not a distraction but an intellectually legitimate response to the complexities inherent in biological systems.

A second camp—articulated with particular force by Fyodor Urnov—contends that the field is at risk of confusing technological novelty with clinical progress. Urnov argues that science is no longer the limiting factor; the tools to treat hundreds of genetic disorders already exist, yet the therapies do not materialize as the prevailing commercial and regulatory climate does not reward their development [138]. The rapid development of a patient-specific base-editing therapy for an infant with CPS1 deficiency showed that a coordinated, integrated workflow can move from diagnosis to first-in-human dosing in months [139,140]. If such a programme is feasible, the persistent lack of treatments for numerous rare diseases appears to reflect institutional limitations rather than merely unresolved technical constraints.

## 6. Lessons for Ophthalmology from Baby KJ

The story of Baby KJ—moving from molecular diagnosis to a patient-specific, in vivo base-editing intervention within months—is a powerful stress test of translational medicine. In a landmark clinical report, Musunuru and colleagues describe the rapid development of a customized lipid nanoparticle-delivered base editor for severe carbamoyl-phosphate synthetase 1 (CPS1) deficiency, followed by infant dosing under regulatory authorization and early biochemical and clinical signals of improved nitrogen handling [140]. Institutional accounts from Children’s Hospital of Philadelphia and the Innovative Genomics Institute demonstrate how the achievement depended on an orchestrated pipeline—patient identification, variant interpretation, editor and guide selection, manufacturing, toxicology, off-target analysis, and bedside delivery—executed as an integrated workflow [141,142].

For ophthalmology, the lesson from this case report is not that the eye should be approached using the same technology as that used in the liver, but that the decisive constraints on genetic therapeutics are increasingly extra-scientific. Fyodor Urnov’s critique, summarized in a 2025 Endpoints News report excerpted by the N = 1 Collaborative, is sharp: for hundreds of monogenic disorders, including many inborn errors of immunity, existing editors and delivery modalities are already adequate; what is missing is the will and structure to deploy them across small patient populations [143]. The technical path from variant identification to a bespoke therapeutic can, in principle, be compressed to weeks, yet remains stymied by economic and regulatory models built for blockbuster drugs. They assume significantly large markets, conventional trial designs and massive clinical trials.

Urnov’s proposed solution is to treat genome editing as a platform technology, with a core set of stable components that include the editor format, delivery vehicle class, manufacturing process, analytics, and standardised workflows for assessing off-target activities. These have already been validated and can be iteratively improved. The programmable component (the guide sequence) varies by patient and genetic target [143,144]. Under conventional regulatory logic, each change in the programmable component is treated as a new drug, reimposing fixed costs that are tolerable for common diseases but prohibitive for rare disorders. Platform thinking does not urge regulators to overlook risk; rather, it asks them to distinguish between what has been tested and derisked and what is actually unique, and to focus scrutiny accordingly.

Encouragingly, regulatory policy is beginning, cautiously, to align with this logic. In May 2024, the U.S. Food and Drug Administration released draft guidance on a Platform Technology Designation Program intended to enable efficiencies in development and review when multiple products share a designated platform [145]. In November 2025, FDA leadership proposed a “plausible mechanism pathway” for bespoke therapies in settings where randomized trials are not feasible, describing a route to marketing authorization based on biological plausibility, small-n clinical evidence, and rigorous post-authorization real-world evidence requirements [146]. Contemporary reporting emphasized that the proposal was motivated by the need for solutions to rare and serious disorders, with Baby KJ serving as a salient example of what can be achieved when we think beyond conventional trial paradigms [147].

Ophthalmology has already seen that regulators can accommodate gene therapies when the benefits are clear and risks are contained. The approval of voretigene neparvovec (Luxturna) for biallelic RPE65-associated retinal dystrophy established a precedent for gene-based treatments for the eye [148]. Yet Luxturna’s success also exposes the inadequacy of a one-disease-at-a-time paradigm: inherited retinal disorders comprise hundreds of genes and a still larger number of pathogenic variants, many too rare to sustain conventional drug development economics [149]. Although the eye offers advantages—surgical access, immune privilege, and non-invasive functional outcome measures—it is not exempt from the system-level problem Baby KJ’s story highlights: without a platform approach, genetic diversity becomes a barrier to treatment rather than an argument for scalable precision medicine.

Baby KJ’s case is a strong argument for building ocular genome-editing platforms rather than accumulating isolated ocular gene therapy products. This involves developing shared editor-and-delivery systems with clear standards; standardized assays for on-target performance, off-target risk, and immunogenicity; and building patient registries designed for real-world data collection rather than passive documentation. Early alignment with regulators to define what constitutes a meaningful change within a platform is required. This is a theme that echoes established European guidance on comparability for advanced therapy medicinal products [150]. The objective is not to diminish standards, but to redirect our emphasis from repeatedly validating the same fundamental elements to continuously learning as we apply a well-governed technology.

The narrative of Baby KJ’s is often framed as a triumph of personalization. However, for ophthalmology, the challenge is to ensure that “personalized” does not mean “artisanal.” Our field’s responsibility is to turn bespoke design into a standardized, repeatable process where a child’s genetic diagnosis initiates a defined, regulated trajectory towards therapy. Baby KJ proves the technology is here today. The remaining question is whether ophthalmology will help build the institutional and regulatory frameworks necessary to standardize these treatments.

## 7. Lessons from a Pivotal Phase III Clinical Trial: Subretinal Timrepigene Emparvovec in Adult Men with Choroideremia

Clinical investigators working in the inherited retinal disease (IRD) domain face substantial structural and regulatory impediments that stem from the stringent visual acuity thresholds imposed by the U.S. Food and Drug Administration (FDA) and the high financial burden associated with late-stage clinical development. The requirement that pivotal trials demonstrate a three-line (15-letter) gain in best-corrected visual acuity (BCVA) on the ETDRS scale constrains enrolment to individuals with sufficiently poor baseline vision to permit detectable improvement [151]. In conditions such as choroideremia, where central visual acuity is typically preserved until later stages of degeneration, this necessitates the recruitment of patients with advanced disease (35–73 ETDRS letters) [48]. This patient sub-group frequently display notable dysfunction and degeneration of the central retinal pigment epithelium (RPE), which is the specific cell targeted for transduction in choroideremia [152,153]. As gene therapy for choroideremia aims to restore REP1 function and prenylation activity to the remaining RPE cells, thereby preventing or slowing RPE cell death, and secondary photoreceptor and choroidal degeneration, dysfunctional RPE may be unable to express the REP1 transgene as efficiently, limiting efficacy and diminishing the likelihood of observing the mandated BCVA gain [154,155]. The consequence is a methodological paradox: the patients most likely to benefit from early intervention are excluded from enrolment, while those who qualify are physiologically less amenable to meeting the regulatory endpoint. In statistical terms, the combination of advanced disease state and high endpoint threshold inflates the sample size required to achieve significance—an expectation realistic in prevalent ocular diseases such as diabetic retinopathy or age-related macular degeneration, but prohibitive in rare IRDs with extremely limited patient pools. Divergent regulatory perspectives further complicate trial design; for example, the European Medicines Agency has regarded a ≥10-letter improvement as clinically meaningful, whereas U.S. approval hinges on the three-line threshold. This discordance enables the possibility that therapeutic eligibility may depend less on biological efficacy than on jurisdictional endpoint selection.

These regulatory hurdles and economic constraints collectively define what is practically achievable in the IRD space. Demonstrating therapeutic benefit in a short pivotal trial—typically one to two years in duration—is intrinsically challenging in diseases characterized by slow, indolent retinal degeneration, for which a decade or more of observation may be required to reveal clinically robust treatment effects. Yet extending trials to biologically appropriate timelines is rendered infeasible by escalating costs, including reliance on contract research organization intermediaries and the financial burden of long-term monitoring. Manufacturing demands further compound the difficulty: the production of adeno-associated viral vectors requires stringent batch validation processes and compliance with globally inconsistent regulatory standards, even though a single 500 mL batch of drug product could treat over 1000 patients with a rare single retinal gene disorder who require 0.1 mL each. The combination of high manufacturing costs, small commercial markets, and rigid endpoint expectations frequently undermines the viability of late-stage development programs. A central challenge in IRD therapeutic translation is not the refinement of vector design or surgical delivery but rather navigating a regulatory and economic environment that structurally disfavours rare disease innovation and, in doing so, risks constraining clinical progress more than the underlying biology itself. These challenges lack the glamour of nuclease refinement, yet they ultimately govern the trajectory of whether an edit becomes a therapy or remains an elegant academic paper.

Currently, the field requires a deliberate shift in investment away from an arms race of editor variants and toward the engineering of translation itself. We should invest in platforms that markets will not naturally build: standardized manufacturing pipelines, regulatory templates, and interoperable clinical registries that can be reused across targets. If gene editing is to fulfill its original promise, coordinated action across academia, industry, and regulatory bodies is needed. It may be that therapies of rare diseases will become an academic exercise rather than a commercial one. The next era of gene editing will be judged less by how many tools we can invent than by how many lives those tools can predictably change.

## Figures and Tables

**Table 2:** Gene editing technologies. Illustrated in Figure 3.

Technology	Mechanism	Key Features	Challenges
CRISPR/Cas9 (DSB-based)	Cas9 nuclease creates DSB at target; repaired by NHEJ or HDR	Versatile; widely used; can disrupt or replace genes	HDR inefficient in non-dividing cells; risk of indels, large deletions and chromosomal translocations; off-target cleavage; immunogenicity
Base editing	dCas9 or Cas9 nickase fused to cytidine or adenine deaminase converts C → T or A → G within editing window	Precise single-base conversion; no DSB; efficient in post-mitotic cells	Limited to specific base changes; editing window leads to bystander edits; deaminase off-target activity; vector size still large
Prime editing	Cas9 nickase fused to reverse transcriptase; pegRNA specifies target and desired edit	Allows all base conversions and small insertions/deletions; no DSB; high precision	Editor (~6.3 kb) exceeds single AAV capacity; requires dual vectors or alternative delivery; editing efficiency modest (~26% in mice); off-target analysis still evolving

## Data Availability

No new data were created or analyzed in this study. Data sharing is not applicable to this article.
